# Longitudinal quantification of choriocapillaris flow deficits in persistent placoid maculopathy: a case report

**DOI:** 10.1186/s12886-023-02894-5

**Published:** 2023-04-18

**Authors:** Jianqing Li, Chris Y. Wu, Mengxi Shen, Leon Bynoe, Joseph Nezgoda, Jeremy Liu, Yuxuan Cheng, Anna Sporysheva, Thomas Albini, Ruikang K. Wang, Giovanni Gregori, Philip J. Rosenfeld

**Affiliations:** 1 Department of Ophthalmology, Bascom Palmer Eye Institute, University of Miami Miller School of Medicine, Miami, FL USA; 2 Department of Ophthalmology, First Affiliated Hospital of Soochow University, Suzhou, Jiangsu China; 3 Retina Associates of Coral Springs, Coral Springs, FL USA; 4 Retina Macula Institute, West Palm Beach, FL USA; 5 Department of Bioengineering, University of Washington, Seattle, WA USA; 6 Department of Ophthalmology, University of Washington, Seattle, WA USA; 7 Bascom Palmer Eye Institute, 900 NW 17th St, Miami, FL 33136 USA

**Keywords:** Persistent placoid maculopathy, Choriocapillaris flow deficits, Optical coherence tomography, Swept source optical coherence tomography angiography

## Abstract

**Background:**

Persistent placoid maculopathy (PPM) is a rare idiopathic chorioretinopathy characterized by choriocapillaris (CC) hypoperfusion. In a case of PPM, we quantified CC flow deficits (FDs) over time and observed an increase in CC perfusion as the visual acuity and outer photoreceptor anatomy improved.

**Case presentation:**

A 58-year-old man was diagnosed with PPM in both eyes based on the patient’s clinical presentation and imaging. He presented with sudden-onset central scotomas in both eyes for about two months. On referral, the best corrected visual acuity (BCVA) was 20/20 in the right eye and 20/100 in the left eye. Plaque-like yellowish macular lesions were observed bilaterally and autofluorescence imaging showed bilateral hyperautofluorescent lesions. Fluorescein angiography (FA) revealed early-phase hyper-fluorescent staining that intensified in the late phases, while indocyanine green angiography (ICGA) displayed persistent hypofluorescence in both eyes. Foveal centered swept source optical coherence tomography (SS-OCT) B-scans showed bilateral focal deposits on the level of retinal pigment epithelium (RPE) and disruption of outer photoreceptor bands. The CC FDs were quantified on SS-OCT angiography (SS-OCTA) images using a previously published algorithm that was validated. The CC FD% was 12.52% in the right eye and 14.64% in the left eye within a 5 mm circle centered on the fovea. After 5 months of steroid treatment, BCVA remained 20/20 in the right eye and improved to 20/25 in the left eye. On OCT imaging, the outer photoreceptor bands fully recovered in both eyes, while some focal deposits remained along the RPE in the left eye. The CC perfusion in both eyes improved, with CC FD% decreasing from 12.52% to 9.16% in the right eye and from 14.64% to 9.34% in the left eye.

**Conclusions:**

Significant impairment of macular CC perfusion was detected after the onset of PPM. Improvement in central macular CC perfusion corresponded with improvements in BCVA and outer retinal anatomy. Our findings suggest that imaging and quantification of CC FDs could serve as a valuable imaging strategy for diagnosing PPM and for following disease progression.

## Background

Persistent placoid maculopathy (PPM) is an idiopathic, bilateral chorioretinopathy that was first described in 2006 [1]. Its pathophysiology has not been clearly established [2]. Previous studies have reported hypoperfusion of the choriocapillaris (CC) in PPM; [3–5] however, CC flow deficits (FDs) have not been quantified previously.

Optical coherence tomography angiography (OCTA) can detect blood flow within the CC by repeating multiple B-scans at a specific position and then comparing differences in the intensity and phase signals from these repeated B scans [6]. These changes in the signals from repeated B-scans are due to the movement of erythrocytes within the capillaries, and result in an angiographic image of blood flow [7–11]. Swept-source optical coherence tomography angiography (SS-OCTA) provides a more detailed visualization of the CC than spectral domain OCT angiography (SD-OCTA) due to its longer wavelength of 1050 nm band compared with the SD-OCTA wavelength of 840 nm band [12, 13]. Due to its longer wavelength band and faster imaging speed, SS-OCTA imaging also allows for better penetration of light through the retinal pigment epithelium (RPE) and denser scan patterns, which results in better detection of blood flow in the CC and improved image quality. Validated algorithms have been developed to quantify the CC flow deficits (FDs) [14].

In this report, we describe a case of PPM with macular CC hypoperfusion at baseline, and as the best-corrected visual acuity (BCVA) improves, the percentage of CC FDs decreases, and the outer photoreceptor anatomy improves.

## Case presentation

A 58-year-old man was referred with a history of sudden-onset central scotomas in both eyes that had persisted for two months. He initially complained of headaches, fevers, chills, and muscle aches for the first five days, and tested negative for COVID-19 twice. His electrocardiogram, chest x-ray, and head computed tomography were normal when performed in the emergency room. There was no remarkable medical or family history. He was referred by an outside retina specialist with a diagnosis of an uncertain maculopathy.

**Fig. 1:**
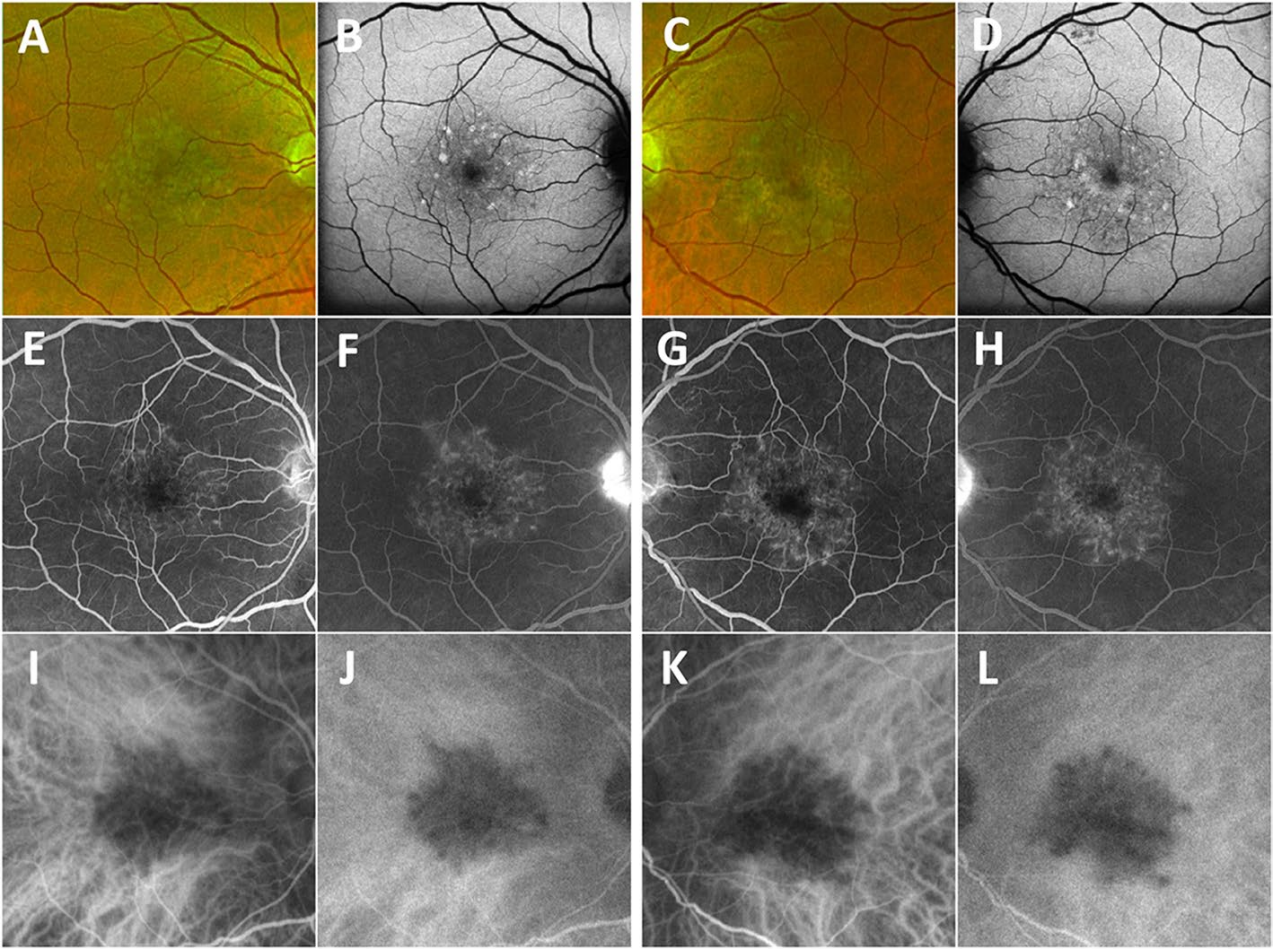
Bilateral fundus appearance at the first visit. **A**, **C** Optos fundus images show plaque-like yellowish lesions in the central maculae of the right (A) and left (C) eyes at presentation. **B**, **D** Autofluorescence of the right (B) and left (D) eyes at presentation show focal hyperautofluorescent lesions. **E**, **F**, **G**, **H**, Early phase **E**, **G** and late phase **F**, **H** fluorescein angiography images of the right **E**, **F** and left **G**. **H** eyes show persistent hyperfluorescence staining that increases in intensity with time. **I**, **J**, **K**, **L** Early phase (I, K) and late phase (J, K) indocyanine green angiography images show persistent hypo-fluorescence in both eyes

**Fig. 2:**
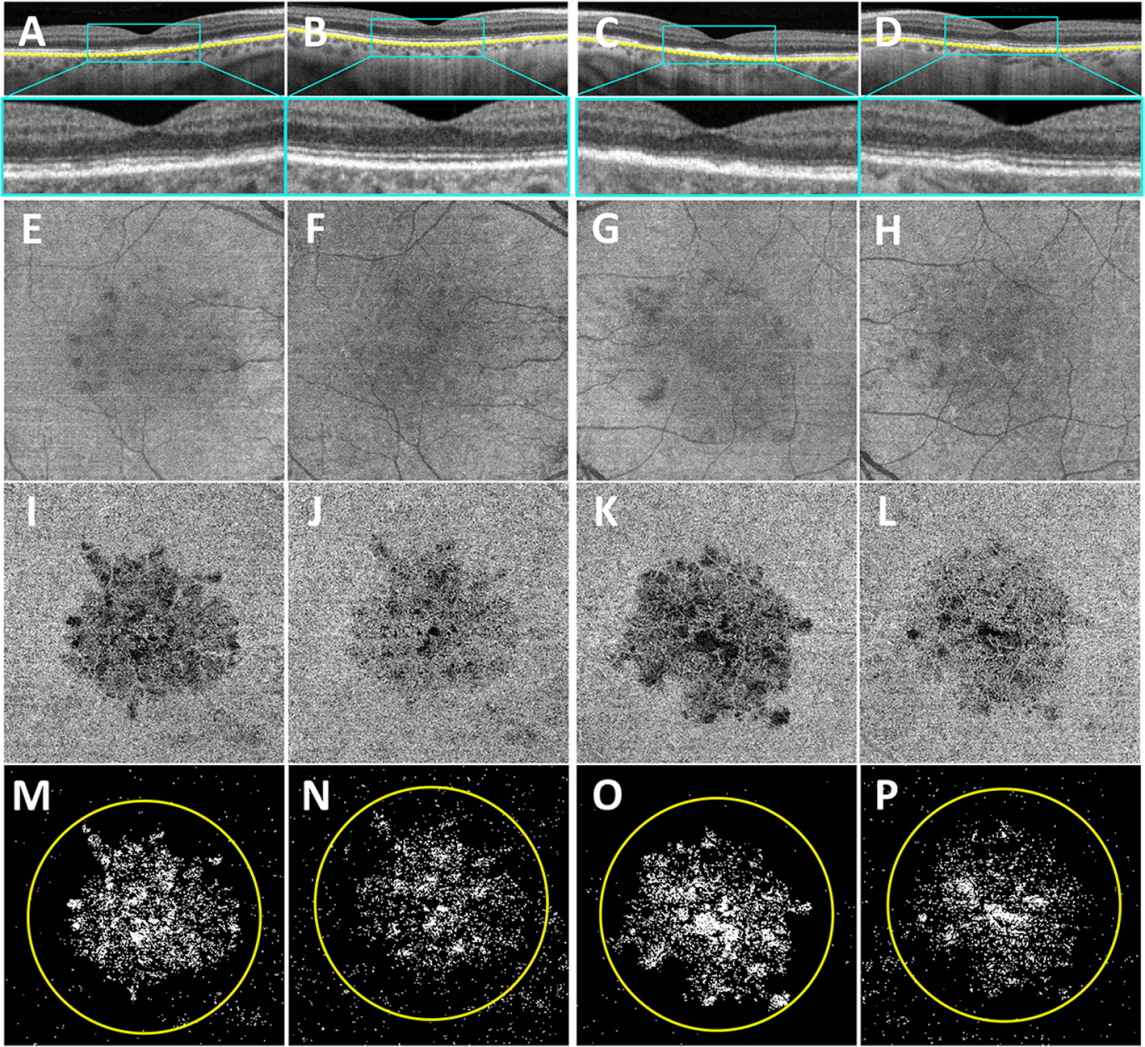
Longitudinal changes in the outer retinal bands and choriocapillaris in both eyes. **A**, **C** The foveal centered SS-OCT B-scans show focal deposits on retinal pigment epithelium (RPE) and disruption of the outer photoreceptor bands in both the right (A) and left (C) eyes at presentation. **B**, **D** 5 months later, the right eye shows restoration of the outer photoreceptors bands (B), while some focal deposits remain along the RPE in the left eye (**D**). The blue boxes show the magnification of the fovea region in both eyes. The yellow lines in A-D were the boundaries of the CC slab. **E**, **G** At the first visit, choriocapillaris (CC) structural images from the algorithm display some focal signal intensity defects in the right (E) and left eyes (G). (F, H) At the 5-month follow-up, CC structural images appeared to have little signal variability throughout the scan area. (I, K) CC flow deficit (FD) images of the right (I) and left eyes (K) show focal areas of CC hypoperfusion at the first visit. (J, L) 5 months later, CC flow images are greatly improved. (M, N, O, P) Binarized CC FDs images with the white denote the area of CC FD. The CC FD% were 12.52% (M) in the right eye and 14.64% (O) in the left eye within a 5 mm circle centered on the fovea at the first visit. At five months of follow-up, the CC FD% were 9.16% (N) in the right eye and 9.34% (P) in the left eyes

On examination, his best corrected visual acuity (BCVA) measurements were 20/20 in the right eye and 20/100 in the left eye. The anterior segment examination was unremarkable in both eyes. Plaque-like yellowish macular lesions were observed bilaterally (Fig. 1A and C). Autofluorescence imaging showed bilateral focal hyperautofluorescent lesions (Fig. 1B and D). Fluorescein angiography (FA) revealed early-phase macular hyperfluorescent staining (Fig. 1E and G) that intensified in the late phases (Fig. 1F and H), while indocyanine green angiography (ICGA) showed persistent macular hypofluorescence in both eyes (Fig. 1I-L). The foveal centered SS-OCT B-scans showed bilateral focal deposits on the retinal pigment epithelium (RPE) and disruption of the outer photoreceptor bands (Fig. 2A and C). The CC FDs were imaged using SS-OCTA and quantified using our previously published method [14]. The CC structural images displayed some focal defects (Fig. 2E and G) while the CC flow images (Fig. 2I-K) showed more obvious foci with loss of perfusion. After binarization, the density of CC FDs within a 5 mm circle centered on the fovea were 12.52% in the right eye and 14.64% in the left eye (Fig. 2M and O). Laboratory tests, which included a complete blood count, angiotensin-converting enzyme levels, a comprehensive metabolic panel, a treponemal syphilis test and a QuantiFERON test, were all found to be unremarkable.

Based on the patient’s clinical presentation and imaging, he was diagnosed with PPM and treated with oral prednisone which started at 60 mg daily and tapered to 10 mg daily over 7 weeks, then subsequently tapered off over 15 weeks. Two months after the onset of treatment, his BCVA remained stable in the right eye at 20/20 and improved to 20/25 in the left eye. At his five-month follow-up visit, the right eye outer photoreceptor bands had fully recovered, while some focal deposits remained along the RPE in the left eye (Fig. 2B and D). Improvements in both the CC structural and flow images were observed (Fig. 2F, H, J and L) with the CC FDs in the right eye decreasing to 9.16%, and in the left eye, the CC FDs decreased to 9.34% (Fig. 2N and P).

## Discussion and conclusions

In this case, significant impairment of macular CC perfusion was detected on the onset of PPM. A previous study argued the choroidal hypoperfusion was due to a masking effect caused by cellular infiltrate, [15] and another research speculated it was an artifact of optical blockage by the placoid lesions [16]. However, our observation that the CC structural images (Fig. 2E-H) had few signal abnormalities while the central CC flow images (Fig. 2I-L) were markedly abnormal suggests that the CC hypoperfusion was real.

In previous reports, CC flow impairment has been suggested based on FA, ICGA and OCTA imaging [1, 3–5, 15, 16]. In this current report, we were able to both document and quantitate CC FDs longitudinally using SS-OCTA. Moreover, CC perfusion improved over time as the visual acuity and the outer retina anatomy improved. At presentation, CC FDs were measured at 12.52% and 14.64% in the right and left eyes respectively, with the better CC perfusion corresponding to better BCVA and outer retinal OCT anatomy in the right eye. After 5 months of treatment, the CC FDs improved bilaterally to about 9%. The photoreceptor and RPE anatomy on OCT imaging returned nearly to normal except for some remaining focal deposits along the RPE in the left eye.

The patient was treated with oral prednisone for 5 months resulting in improved vision and no evidence of macular neovascularization, which had been reported previously [3]. Although oral steroids are generally used as first-line agents, [2] it remains controversial whether steroids are needed or if gradual improvement is the normal course of disease progression.

This report demonstrates the value of SS-OCTA imaging for both the structural integrity of the outer retina and RPE as well as the quantitation of central macular CC FDs. We then correlated these findings with disease symptoms and were able to show that CC perfusion improved as BCVA improved and the outer retinal OCT anatomy was restored. These results suggest that imaging and quantitation of CC FDs should be a valuable parameter to monitor when following disease progression in PPM.

## Data Availability

All data supporting our findings are provided in the manuscript.

## Abbreviations

PPM: persistent placoid maculopathy
CC: choriocapillaris
CC FDs: choriocapillaris flow deficits
BCVA: best corrected visual acuity
FA: fluorescein angiography
ICGA: indocyanine green angiography
OCT: optical coherence tomography
SS-OCT: swept source optical coherence tomography
OCTA: optical coherence tomography angiography
SS-OCTA: swept source optical coherence tomography angiography
SD-OCTA: spectral domain optical coherence tomography angiography
RPE: retinal pigment epithelium
